# Imposthume

**Published:** 1858-06

**Authors:** 


### IMPOSTHUME.

FIFTY-NINE years ago, the great WASHINGTON wrote to his sister, of his recovering from a "long and tedious complaint," by the doctors making an "incision for the cure of an *Imposthume*," a kind of abscess or boil on his thigh. Within a year, we have seen it announced in a British medical publication, with an intimation of its novelty, that the speediest method of getting rid of a boil is, to make a deep crucial incision—that is, cut it crossways to the very core, with a lancet. A great deal of trouble is taken generally to "bring it to a head," by poulticing it with every variety of things, clean and filthy, namable and unnamable; meanwhile, an immense amount of suffering is endured, with an infinitesimal amount of patience. To such as have decision and courage, we say, a boil is nipped in the bud, and is completely cured, in a very short time, if it is cut down deeply with a lancet, first in one direction, and then at right angles to it, by giving exit to the blood at once, which, if left there, keeps up the heat and pain until it is converted into yellow matter, and is pushed out of the system by force. So it is better to help Nature with a lancet, let the blood out at once. and be done with it.

